# Segurança dos Procedimentos da Cardiologia Intervencionista na Síndrome Coronariana Crônica durante a Pandemia de COVID-19

**DOI:** 10.36660/abc.20200704

**Published:** 2020-10-13

**Authors:** Esmeralci Ferreira, Thales Siqueira Alves, Ricardo Mourilhe-Rocha, Ana Luiza Iannarella Lacerda, Felipe Neves Albuquerque, Pedro Pimenta de Mello Spineti, Daniel Xavier de Brito Setta, Roberto Esporcatte, Denilson Campos Albuquerque

**Affiliations:** 1 Universidade do Estado do Rio de Janeiro Rio de JaneiroRJ Brasil Universidade do Estado do Rio de Janeiro, Rio de Janeiro, RJ – Brasil

**Keywords:** Pandemia, SARS-CoV-2, Betacoronavírus, Doença da Artéria Coronariana/mortalidade, Intervenção Coronária Percutânea, Perfil de Saúde

A doença cardiovascular (DCV) é a principal causa de mortalidade no Brasil e no mundo, o que implica aumento de morbidade, com seus fatores de risco bem definidos. A doença coronariana aguda tem as indicações de tratamentos intervencionistas muito bem estabelecidas, e nas síndromes coronarianas crônicas (SCC), as indicações de intervenção são pautadas no grau de isquemia e na sintomatologia dos pacientes.^1^^,^^2^ No entanto, a pandemia de COVID-19 causada pelo novo Coronavírus, mutação do vírus que provoca a síndrome respiratória aguda grave (SARS-CoV-2), mudou de forma radical as indicações de procedimentos intervencionistas, independentemente da apresentação clínica.^3^

A ampla transmissão comunitária, o grave acometimento e a complexidade da doença resultam em uma taxa de mortalidade que pode chegar a 12% nos grupos de risco, principalmente portadores de DVC.^3^ Por isso, a COVID-19 desencadeou uma mudança de paradigma nos atendimentos cardiológicos em todo o mundo, principalmente nos ambientes da cardiologia intervencionista.^4^^-^^8^

Nas síndromes coronarianas agudas (SCA) houve uma expressiva redução da procura dos pacientes pelas salas de emergência, talvez por medo da infecção ou mesmo por estarem menos sintomáticos no período de confinamento. Retardos no sistema de regulação dos serviços públicos também ocorreram, certamente provocados por sobrecarga na internação hospitalar.^6^ Por outro lado, os setores de cardiologia intervencionista restringiram os atendimentos a esses pacientes, e novas rotinas foram criadas para realização de intervenções, apenas em situações de maior gravidade, com verdadeira desmobilização nos protocolos de dor torácica.^7^^,^^8^ Por exemplo, muitos hospitais terciários preconizaram a indicação de trombólise, em vez de angioplastia primária, e outros realizaram procedimentos intervencionistas apenas mediante testes rápidos para excluir a possibilidade de infecção pelo SARS-CoV-2. Todas essas ações foram respaldadas ou orientadas pelas sociedades de cardiologia intervencionista do mundo inteiro.^4^^,^^5^^,^^7^^,^^8^

Nas SCC, os exames de diagnóstico invasivo foram literalmente suspensos para pacientes eletivos, sem previsão de agendamento. Isso ocorreu no Sistema Único de Saúde (SUS), cuja suspensão das consultas eletivas também impactou de forma indireta na redução dos procedimentos. No sistema suplementar de saúde, as senhas de autorização foram suspensas por diversas seguradoras.

Admitir que pacientes com SCC apresentem menor gravidade não condiz com os dados observados em literatura, os quais demonstram incidência de lesões obstrutivas significativas em mais de 50% dos casos.^9^^-^^11^ Em contrapartida, não há dados de literatura sugerindo a realização de procedimentos de intervenção em indivíduos estáveis durante a pandemia. Partindo da premissa de que, nas SCC, os procedimentos não devem ser adiados, uma vez que os pacientes são potencialmente portadores de coronariopatia grave, foi organizada uma coorte de pacientes para efetiva orientação por meio de consultas. Independentemente da fase de afastamento social imposta pela pandemia, os procedimentos foram realizados da forma mais segura possível. O objetivo principal foi avaliar, em uma população inicial de 105 pacientes do SUS com SCC, se havia segurança em relação ao risco de infecção pelo SARS-CoV-2 na realização de coronariografia com ou sem intervenção coronária percutânea (ICP). Foram analisados: perfil clínico, resultado angiográfico, necessidade de revascularização, mortalidade e ocorrência ou não de suspensão de exames devido a diagnóstico ou suspeição da infecção.

## Métodos

Neste estudo prospectivo realizado durante a pandemia de COVID-19, foram avaliados 105 pacientes do SUS com SCC por meio de coronariografia eletiva, atendidos em um hospital universitário entre março e maio de 2020. Quatro deles foram excluídos por não comparecerem ao exame na data agendada. Todos foram avaliados previamente em consulta médica com um cardiologista, havendo inclusão de dados do perfil clínico de cada um. Durante a consulta e assinatura do termo de consentimento livre e esclarecido, os pacientes foram orientados a fazer isolamento social, o que foi entendido por todos. Os procedimentos foram realizados de forma segura com equipamentos de proteção individual (EPI) tanto para as equipes quanto para os pacientes. As condutas foram tomadas com base nas lesões coronarianas.

Foram consideradas graves aquelas acima de 70% do lúmen, em vasos epicárdicos e acima de 50% em tronco de coronária esquerda (TCE). As lesões foram avaliadas por dois ou mais observadores experientes. A avaliação da sintomatologia clínica para a presença de COVID-19 foi feita durante a consulta, no período hospitalar e após 15 dias de qualquer presença no hospital. A orientação para realização de teste foi programada apenas em caso de suspeição da doença. O estudo foi aprovado pelo comitê de ética e pesquisa da instituição (CAAE 31784420.7.0000.5259. Número do Parecer: 4.035.853)

### Análise estatística

Os dados foram analisados por meio do software da IBM SPSS, versão 25.0. As variáveis contínuas foram descritas com base em sua média e em seu desvio-padrão; e as variáveis categóricas, de acordo com esse número absoluto em percentual.

## Resultados

Entre março e maio de 2020, 194 consultas ambulatoriais pré-cateterismo foram realizadas, e 105 pacientes tiveram seus exames agendados. Dois deles (1,03%) apresentaram síndrome gripal na data marcada e, por isso, foram orientados a manter isolamento domiciliar por 15 dias e procurar hospital em caso de piora. Ambos faltaram na data do exame; logo, não se obteve informação posterior sobre a evolução dos mesmos. Outros dois pacientes faltaram ao exame e não houve mais contato. Cento e um pacientes compareceram para realização do procedimento, mas um deles evoluiu com óbito cardiovascular antes (taquicardia ventricular). Foram considerados 101 indivíduos analisados, com a realização de cateterismo associado ou não à ICP em 100 pacientes e 15 (14,8%) internações para o procedimento. Ocorreram 11 ICP e três cirurgias de revascularização do miocárdio (CRVM). A média de idade foi 61,88 ± 10,3 anos, e 51,5% eram homens. Dentre os fatores de risco para doença arterial coronariana (DAC), a hipertensão arterial sistêmica (HAS), o diabetes melito (DM) e a dislipidemia (DLP) foram os mais prevalentes (Tabela 1).

**Tabela 1:** Características gerais da população avaliada

Características de base		Pacientes analisados (n = 101)
Idade (anos)		61,88 ± 10,3
Masculino		52 (51,5)
Feminino		49 (48,5)
Tabagismo		19 (18,8)
Hipertenso		89 (88,1)
Diabetes melito		41 (40,6)
Dislipidemia		31 (30,7)
IAM prévio		31 (30,7)
CAT prévio		8 (7,9)
CRVM prévia		7 (6,9)
Apresentação clínica		
AE		101(100)
TNI		
Realizados		37 (37)
	DAC obst.	19 51,4
	Sem DAC obst.	18 48,6
Não realizados		63 (63)
	DAC obst.	35 (55,6)
	Sem DAC obst.	28(44,4)

Valores demonstrados em n (%). IAM: infarto agudo do miocárdio; CAT: cateterismo coronariano; CRVM: cirurgia de revascularização do miocárdio; AE: angina estável; TNI: teste não invasivo; DAC: doença arterial coronariana; Obst.: obstrutiva.

**Figura 1:**
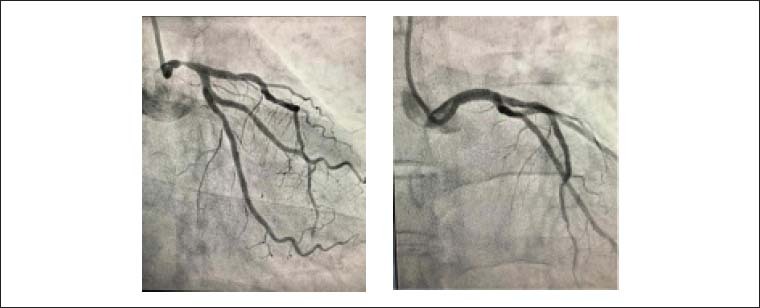
Coronariografia em duas projeções mostrando lesão de 75% no tronco da coronária esquerda (TCE) e pós-implante de stent farmacológico de 4,0 x 12 mm no TCE.

A prevalência de DAC obstrutiva foi encontrada em 54%, sendo que 22% apresentavam acometimento trivascular, com envolvimento de 8% no TCE e 35% na artéria descendente anterior (ADA) (Tabela 2). Nos pacientes com envolvimento de TCE, 87,5% estavam associados à DAC multiarterial, e somente um paciente apresentou estenose isolada em corpo de TCE (Figura 1). Dentre os homens, 66,6% apresentaram DAC obstrutiva e 40,8% entre as mulheres. A DAC ocorreu em 63% dos pacientes com idade superior a 60 anos. A via radial foi utilizada em 97% dos casos. ICP ou CRVM de urgência foi feita em 14% dos pacientes com DAC obstrutiva. Dentre as ICP realizadas, 70% trataram apenas um vaso.

**Tabela 2:** Características angiográficas e condutas

Características angiográficas		Procedimentos (n = 100)
CAT		89
CAT e ICP		11
	Ad hoc	4 (36,4%)
	Urgente	7 (63,6%)
CRVM		3
Óbito		1
	Uniarterial	20
	Biarterial	12
	Triarterial	22
**Localização das lesões nas artérias**		
	TCE	8
	DA	35
	CX	32
	CD	32
**Vias de acesso**		
	Radial	97
	Femoral	3

CAT: cateterismo coronariano; ICP: intervenção coronariana percutânea; CRVM: cirurgia de revascularização do miocárdio; TCE: tronco de coronária esquerda; DA: descendente anterior; CX: circunflexa; CD: coronária direita.

Entre os oito examinadores e membros da equipe, nenhum apresentou suspeita/confirmação de COVID-19 durante o período do estudo, e nenhum dos pacientes internados mostrou sintomas clínicos de COVID-19 durante a internação. Todos os que realizaram procedimentos se mantiveram em isolamento conforme orientação prévia. Em relação àqueles que fizeram o procedimento, nenhum apresentou síndrome gripal nos 15 dias posteriores ao exame.

## Discussão

Este estudo preliminar mostrou segurança para pacientes e equipe na realização de exames eletivos, mesmo durante a pandemia. No entanto, não há dados comparativos, pois não existem dados na literatura avaliando esse tipo de população. Analisando todos os pacientes dessa casuística, foi observado que, a despeito do quadro de SCC, um percentual significativo (14,8% dos pacientes) apresentou eventos graves, com um óbito, 14 indicações a internação para revascularização imediata (*ad hoc*) ou dentro da primeira semana após o procedimento, sendo três com encaminhamento para cirurgia. A média de idade de 61,8 anos e a maior incidência de hipertensão e diabetes melito foram semelhantes aos dados de outros estudos em pacientes submetidos a coronariografia.^9^^,^^10^

A taxa de exames normais (46%) foi dentro daquela encontrada na literatura, conforme estudo de Costa et al.,^9^ o qual avaliou uma coorte de 1.844 pacientes submetidos à coronariografia. Os autores também enfatizaram um percentual significativo (52,9%) de DAC grave nas SCC.

Sant’Anna et al. relataram 45% de coronárias normais em uma avaliação de 503 pacientes, mas a prevalência era em população constituída de pacientes jovens, do sexo feminino e não tabagistas.^10^ Já um estudo americano^11^ com 1.989.779 exames encontrou uma prevalência de DAC moderada/grave de 41,0% em pacientes com SCC. Analisando indivíduos com DAC obstrutiva, 26% tiveram necessidade de intervenção de urgência devido à gravidade das lesões.

Apesar de ter ocorrido um óbito antes do exame, a presença de lesão de tronco em apenas 8% dos pacientes e de envolvimento triarterial em 22% em relação a indivíduos estáveis superou as expectativas. A gravidade das lesões de TCE também não correspondeu ao que se costuma encontrar em pessoas com SCC, com somente um paciente tratado de forma urgente, por via percutânea (Figura 1). Cerca de 74% dos pacientes com indicação de CRVM ou ICP foram orientados para que seus procedimentos fossem agendados de forma eletiva. Dentre aqueles com DAC obstrutiva (54%), somente 26% (14) já realizaram procedimento de revascularização, e 74% (40) estavam com anatomia coronariana obstrutiva conhecida aguardando procedimento.

Nas SCA, as orientações para atendimento durante a pandemia são mais bem estabelecidas, inclusive com respaldo de várias sociedades de cardiologia, sempre respeitando o equilíbrio entre a exposição da equipe e o benefício do paciente.^3^^,^^4^^,^^6^^,^^7^ No infarto agudo do miocárdio com supradesnível do segmento ST (IAMCSST) em pacientes com COVID-19 ativo, a fibrinólise pode ser considerada uma opção em indivíduos relativamente estáveis.^3^^-^^6^ Nos instáveis ou com potencial deterioração clínica, a ICP primária (ICPP) deve ser realizada.

Em um estudo multicêntrico nacional desenvolvido na Itália, envolvendo 54 hospitais durante o período de uma semana comparativa, entre os anos de 2019 e 2020, houve uma redução significativa de 48,4% (319 x 618, p < 0,001) na quantidade de infartos; entretanto, o número de casos fatais aumentou para 13,7%, comparados aos 4,1% previamente registrados em 2019 (RR = 3,3; 95% IC 1,7 a 6,6; p < 0,001).^12^ O registro de Nova Iorque (EUA) também evidenciou aumento de mortalidade em domicílios cerca de 8 a 10 vezes em relação ao mesmo período em anos anteriores.^13^ Esses resultados estão alinhados com o registro espanhol,^14^ que mostra redução de 40% dos casos de IAMCSST, e em diferentes estados norte-americanos cuja redução nas internações variou entre 38 e 48%.^13^

No infarto agudo do miocárdio sem supradesnível do segmento ST (IAMSSST), os relatos também apontam para uma diminuição acentuada no número das internações semanais, como avaliado no estudo italiano, sendo de 350 para 122 (65,4% de redução; 95% IC 60,3 a 70,3; p < 0,001).^12^ A recomendação mais aceita no IAMSSST é que, se houver possibilidade, a realização de testes para COVID-19 antes do cateterismo cardíaco é uma boa alternativa, sendo que pacientes mais graves deverão ser submetidos à intervenção precocemente.^3^^,^^4^^,^^6^^,^^7^

Nas SCC, as recomendações de intervenções são menos consistentes e consideram de forma superficial que as condutas devam ser individualizadas e indicadas apenas em pacientes de alto risco.^6^^,^^8^ Welt et al.^8^ preconizam a redução do número de procedimentos, com adiamento de casos eletivos e divisão da equipe em turnos para rodízio de profissionais, com ênfase na redução do risco de contaminação da equipe.

Essas medidas restritivas relacionadas aos pacientes estáveis proporcionaram uma zona de conforto em relação à redução da propagação da COVID-19; entretanto, de maneira objetiva, observou-se que a atenção à doença coronariana não estava contemplada no que se refere às boas práticas previamente estabelecidas antes da pandemia.

Em nosso meio, a pandemia alterou de maneira contundente o atendimento médico em diversas especialidades, com suspensão de procedimentos e consultas eletivas. A maioria da classe médica orientou, tanto a pacientes oriundos do sistema de saúde suplementar quanto aos usuários do SUS, a protelarem seus procedimentos eletivos e a recorrerem às salas de emergência em casos de sintomas intensos de precordialgia, dispneia etc. Essa orientação, sem dúvida, protege do novo Coronavírus (SARS-CoV-2) os indivíduos que se mantiverem assintomáticos ou oligossintomáticos, mas expõe à infecção aqueles que, em casos de necessidade premente, necessitem do atendimento. Por outro lado, indivíduos que não julguem sua sintomatologia tão intensa ou que sejam mais tolerantes à dor podem ser acometidos de uma reflexão temerária. Nesses casos, o adiamento da procura por socorro pode ter consequências graves, inclusive a de morte cardíaca domiciliar.

A resolução nº 2004, de 18/03/2020, da Secretaria Estadual de Saúde do Rio de Janeiro (SES-RJ)^15^ suspendeu os atendimentos ambulatoriais eletivos nas unidades públicas; porém, de maneira acertada, manteve os atendimentos ambulatoriais essenciais, incluindo os de oncologia e cardiologia. Mesmo assim, o acesso dos pacientes foi afetado, e a resolução não foi acatada de forma ampla. Via de regra, os pacientes do SUS são mais graves e com mais fatores de riscos. Nestes, há grandes dificuldades em descobrir quem realmente é estável do ponto de vista global e quem pode ser acometido por uma agudização do quadro, com necessidade de internação. Por isso, parece razoável preservar a capacidade dos leitos hospitalares, evitando procedimentos eletivos desnecessários em pacientes estáveis, com comorbidades significativas ou nos quais o tempo de internação pós-intervenção seja superior a 24 a 48 horas. Não obstante, a despeito do alinhamento com a maioria das medidas protetivas para pacientes e equipe, ressalta-se que, em pacientes com SCC, existe a possibilidade de realização dos procedimentos e da quebra cautelosa nas medidas restritivas, sem riscos adicionais de exposição ao vírus.

Na série de casos apresentada, a busca ativa foi feita procurando selecionar os sintomáticos, isquêmicos e com múltiplos fatores de risco, evitando, assim, que eles procurassem qualquer tipo de consulta ou ida a um hospital de emergência. Entende-se que essa ação rápida no diagnóstico e tratamento de tais pacientes foi uma medida que evitou um desfecho mais grave em termos de eventos coronarianos. Por outro lado, os cuidados rígidos e as orientações que ocorreram desde a consulta, bem como o reforço de isolamento, as rotinas no procedimento e a tentativa de reduzir ao máximo o tempo de internação minimizou os riscos de infecção pelo novo Coronavírus. Na crise da saúde desencadeada pela COVID-19, não houve orientações objetivas sobre a realização dos procedimentos em pacientes estáveis, exatamente em função de suspensão ou adiamento dos mesmos. Na experiência “de mundo real”, o atendimento dos pacientes com suspeita de DAC por meio de uma avaliação clínica e do conhecimento rápido da anatomia coronariana deve ser feito antes de uma possível instabilidade clínica. Isso porque, nessa população, demonstra-se de forma clara que o risco de eventos cardíacos aos quais estão suscetíveis é muito maior do que a possibilidade de apresentar complicações da doença causada pelo vírus.

Embora não seja determinado que essa seja efetivamente a melhor conduta, os dados sugerem uma reflexão para que os atendimentos nos laboratórios de hemodinâmica, nesta fase de pandemia, sejam reavaliados e não sejam suspensos ou adiados de forma sistemática.

## Limitações

As principais limitações são o número pequeno de pacientes, as variáveis de baixa prevalência que necessitam de amostras muito maiores e o caráter unicêntrico do estudo. Além disso, por orientação da Comissão de Controle de Infecção Hospitalar, não foram realizados testes para COVID-19 em nenhum dos pacientes e na equipe, pois todos estavam assintomáticos no momento do procedimento e permaneceram assim por no mínimo 15 dias. Apesar das limitações, as observações podem servir de estímulo para que outros serviços desenvolvam análises multicêntricas mais robustas estatisticamente.

## Conclusões

Nosso ponto de vista é pautado neste estudo, no qual a realização dos exames eletivos em indivíduos com doença coronariana crônica foi segura para pacientes e profissionais, mesmo durante a pandemia, sendo um contraponto à maioria das recomendações de outros serviços. O estudo possibilitou demonstrar que a avaliação angiográfica anatômica revelou pacientes de alto risco de morbimortalidade, com necessidade de intervenções naqueles com lesões complexas. Isso contribuiu para reduzir o número de síndromes coronarianas agudas nessa população.
