# Supplementary material for: Segurança dos Procedimentos da Cardiologia Intervencionista na Síndrome Coronariana Crônica durante a Pandemia de COVID-19
Source: Arq Bras Cardiol. 2020 Oct 13;115(4):712–6. [Article in Portuguese] doi: 10.36660/abc.20200704 (PMC8386985; doi:10.36660/abc.20200704)
Supplement: Translation

# Translation: Safety of Interventional Cardiology Procedures in Chronic Coronary Syndrome during the COVID-19 Pandemic

**Authors:** Esmeralci Ferreira, Thales Siqueira Alves, Ricardo Mourilhe-Rocha, Ana Luiza Iannarella Lacerda, Felipe Neves Albuquerque, Pedro Pimenta de Mello Spineti, Daniel Xavier de Brito Setta, Roberto Esporcatte, Denilson Campos Albuquerque

**Affiliations:** 1 Rio de JaneiroRJ Brazil Universidade do Estado do Rio de Janeiro, Rio de Janeiro, RJ – Brazil

**Keywords:** Pandemics, SARS- CoV-2, Betacoronavirus, Coronary Artery Disease/mortality, Percutaneous Coronary Intervention, Health Profile

Cardiovascular disease (CVD) is the leading cause of mortality in Brazil and in the world, with increased morbidity and well-defined risk factors. Acute coronary heart disease has very well-established indications for interventional treatments. In chronic coronary syndrome (CCS), the indications for intervention are based on the degree of ischemia and the symptoms of each patient.^1^^,^^2^ The pandemic triggered by severe acute respiratory syndrome coronavirus 2 (SARS-CoV-2), which causes COVID-19, has radically changed the indications for interventional procedures, regardless of clinical presentation.^3^

Wide community transmission, severe involvement and complexity of the disease, and a mortality rate that may reach up to 12% in risk groups, mainly those with CVD, have been observed.^3^ COVID-19 has triggered a paradigm shift in cardiology care worldwide, especially in interventional cardiology settings.^4^^-^^8^

In acute coronary syndrome (ACS), there has been a significant reduction in the number of patients seeking emergency rooms, perhaps due to fear of infection or even because they were less symptomatic during lockdown. Delays in the public service regulation system have also occurred, certainly caused by overload in hospital admissions.^6^ Conversely, interventional cardiology departments have restricted care to these patients, and new routines have been created to perform interventions only in more severe situations, with real demobilization in chest pain protocols.^7^^,^^8^ Many tertiary care hospitals have recommended thrombolysis rather than primary angioplasty, and others have performed interventional procedures only after rapid testing to exclude SARS-CoV-2 infection. All actions have been supported or guided by interventional cardiology societies worldwide.^4^^,^^5^^,^^7^^,^^8^

Invasive diagnostic tests have been fully suspended for elective patients with CCS, with no schedule for appointments. This has occurred in the Brazilian Unified Health System (SUS), whose suspension of elective appointments has also had an indirect impact on the reduction in procedures. In the private health insurance system, authorization passwords have been suspended by several insurers.

Acknowledging that patients with CCS are less severe does not represent the data seen in the literature, which demonstrate incidence of significant obstructions in more than 50% of cases.^9^^-^^11^ In contrast, we found no data suggesting interventional procedures for stable individuals during the pandemic. Based on the assumption that procedures in patients with CCS should not be delayed, as they potentially have severe coronary heart disease, we organized a cohort of patients to conduct effective guidance during appointments. Regardless of the stage of social distancing imposed by the pandemic, the procedures were conducted in the safest way possible. Our primary objective was to evaluate whether performing coronary angiography with or without percutaneous coronary intervention (PCI) was safe regarding the risk of SARS-CoV-2 infection in an initial population of 105 SUS patients with CCS. We analyzed clinical profile, angiography results, need for revascularization, mortality, and whether tests were suspended due to diagnosis or suspicion of the infection.

## Methods

In this prospective study conducted during the COVID-19 pandemic, 105 SUS patients with CCS undergoing elective coronary angiography at a teaching hospital between March and May 2020 were evaluated. Four patients were excluded for not attending the test on the scheduled date. All patients were previously evaluated by a cardiologist in a medical appointment, including data on each patient’s clinical profile. During the appointment and when signing the informed consent form, patients were advised to make social isolation, and the guidance was understood by all. The procedures were performed safely with both staff and patients wearing personal protective equipment (PPE). The approaches were taken based on the coronary lesions. Those above 70% of the lumen in epicardial coronary vessels and those above 50% in the left main coronary artery (LMCA) were considered severe. The lesions were assessed by two or more experienced observers. The assessment of clinical symptoms for presence of COVID-19 was made during the appointment, in the hospital stay, and after 15 days of being at the hospital. The guidance for performing the test was scheduled only in case of suspicion of the disease. The study was approved by the institution’s research ethics committee (registration number: CAAE 31784420.7.0000.5259; opinion number: 4.035.853).

### Statistical analysis

Data were analyzed using IBM SPSS, version 25.0. Continuous variables were described as mean and standard deviation, and categorical variables were described as absolute numbers and percentages.

## Results

In total, 194 precatheterization outpatient appointments were made and 105 patients had their tests scheduled between March and May 2020. With regard to appointments, two patients (1.03%) had flu-like syndrome on the day of the appointment and were advised to maintain isolation at home for 15 days and to seek care at the hospital if their condition deteriorated. Both patients did not attend the scheduled test and no further information was obtained about their progress. Two other patients missed the exam and there was no further contact. Onde hundred and one patients attended the test. One patient had cardiovascular death before the procedure (ventricular tachycardia). We considered 101 patients for analysis, including 100 patients undergoing catheterization either combined with PCI or not and 15 (14.8%) admissions for the procedure. There were 11 PCIs and 3 coronary artery bypass grafts (CABGs). Mean age was 61.88 ± 10.3 years, and 51.5% were male. Hypertension, diabetes mellitus, and dyslipidemia were the most prevalent risk factors for coronary artery disease (CAD) (Table 1).

**Table 1:** General characteristics of the population

Baseline characteristics		Patients analyzed (N = 101)
Age (years)		61.88 ± 10.3
Male		52 (51.5)
Female		49 (48.5)
Smoking		19 (18.8)
Hypertension		89 (88.1)
Diabetes mellitus		41 (40.6)
Dyslipidemia		31 (30.7)
Previous AMI		31 (30.7)
Previous Cath		8 (7.9)
Previous CABG		7 (6.9)
Clinical presentation		
SA		101 (100)
NIT		
Performed		37 (37)
	Obst. CAD	19 51.4
	No obst. CAD	18 48.6
Not performed		63 (63)
	Obst. CAD	35 (55.6)
	No obst. CAD	28 (44.4)

Values shown as n (%). AMI: acute myocardial infarction; CABG: coronary artery bypass graft; CAD: coronary artery disease; Cath: coronary catheterization; NIT: noninvasive test; Obst.: obstructive; SA: stable angina.

The prevalence of obstructive CAD was 54%, of which 22% had triple-vessel involvement, with 8% involving the LMCA and 35% involving the left anterior descending artery (LAD) (Table 2). In patients with LMCA involvement, 87.5% were associated with multivessel CAD and only one patient had isolated stenosis in the LMCA (Figure 1). Obstructive CAD was found in 66.6% of men and 40.8% of women. CAD occurred in 63% of patients aged > 60 years. Radial access was used in 97% of cases.

**Table 2:** Angiographic characteristics and approaches

Angiographic characteristics		Procedures (N = 100)
Cath		89
Cath and PCI		11
	Ad hoc	4 (36.4)
	Urgent	7 (63.6)
CABG		3
Death		1
	Single-vessel	20
	Double-vessel	12
	Triple-vessel	22
Location of lesions in the arteries		
	LMCA	8
	LAD	35
	LCX	32
	RCA	32
Access routes		
	Radial	97
	Femoral	3

Values shown as n (%). CABG: coronary artery bypass graft; Cath: coronary catheterization; LAD: left anterior descending artery; LCX: left circumflex artery; LMCA: left main coronary artery; PCI: percutaneous coronary intervention; RCA: right coronary artery.

**Figure 1:** coronary angiography in two projections showing a 75% lesion in the left main coronary artery (LMCA) and then following 4.0 x 12 mm drug-eluting stent implantation in the LMCA.

PCI or urgent CABG was performed in 14% of patients with obstructive CAD. Of all PCIs performed, 70% treated only one vessel.

Among the eight examiners and staff members, none had suspected or confirmed COVID-19 during the study. None of the admitted patients had COVID-19 symptoms during hospitalization. All patients who underwent procedures were kept in isolation according to previous guidance. Regarding the patients who underwent the procedure, none had flu-like syndrome within 15 days of the test.

## Discussion

This preliminary study showed safety for both patients and staff in conducting elective tests, even during the pandemic. We do not have comparative data because there are no studies in the Brazilian literature evaluating this type of population. By evaluating all the patients in this series, we observed that, despite CCS, a significant percentage of 14.8% of the patients had serious events, including 1 death and 14 indications for admission for immediate (ad hoc) revascularization or within the first week following the procedure, 3 of which were indicated for surgery. The mean age of 61.8 years and the higher incidence of hypertension and diabetes mellitus were similar to those of other studies of patients undergoing coronary angiography.^9^^,^^10^

The rate of normal test results (46%) was within that found in the literature according to a study conducted by Costa et al.,^9^ who investigated a cohort of 1844 patients undergoing coronary angiography. Those authors also found a significant percentage (52.9%) of severe CAD in CCS.

Sant’Anna et al. reported 45% of normal coronary arteries in an evaluation of 503 patients, but the prevalence was found in a population consisting of young, female, nonsmoker patients.^10^ An American study^11^ of 1 989 779 tests found a prevalence of moderate-to-severe CAD of 41.0% in patients with CCS. Regarding patients with obstructive CAD, 26% required urgent intervention because of the severity of the lesion.

The presence of LMCA disease (8%) and triple-vessel involvement (22%) exceeded expectations for stable patients, as well as the occurrence of 1 death before the test. Also, the severity of LMCA disease does not correspond to that found in patients with CCS, and 1 patient was treated urgently by percutaneous route (Figure 1). Approximately 74% of patients with indication for CABG or PCI were advised to have their procedures scheduled electively. Among those with obstructive CAD (54%), only 26% (14) have already undergone a procedure for revascularization, and 74% (40) have known obstructive coronary anatomy awaiting a procedure.

The guidelines for ACS care during the pandemic are better established, with the support of several cardiology societies, always respecting the balance between staff’s exposure and patient’s benefit.^3^^,^^4^^,^^6^^,^^7^ In cases of ST-segment elevation myocardial infarction (STEMI) and active COVID-19, fibrinolysis may be considered an option in relatively stable patients.^3^^-^^6^ In unstable patients or those with potential clinical deterioration, primary PCI should be performed. In a national multicenter study involving 54 hospitals in Italy, there was a significant reduction of 48.4% (319 x 618, p < 0.001) in the number of infarctions during a comparative week between 2019 and 2020; however, the number of fatal cases increased by 13.7% compared to 4.1% that was previously recorded in 2019 (RR = 3.3, 95% CI 1.7–6.6; p < 0.001).^12^ A New York (United States, US) registry also showed increased mortality in households, about 8-10 times, compared to the same period in previous years.^13^ These results are consistent with those of a Spanish registry^14^ that showed a 40% reduction in STEMI cases and those of different US states whose reduction in admissions ranged from 38 to 48%.^13^

In non-ST-segment elevation myocardial infarction (NSTEMI), the reports also demonstrated a sharp decrease in the number of weekly admissions, as shown in the Italian study, in which the number reduced from 350 to 122 (65.4% reduction; 95% CI 60.3–70.3; p < 0.001).^12^ The most accepted recommendation for NSTEMI is, if possible, performing COVID-19 tests before cardiac catheterization, and more severe patients should undergo early intervention.^3^^,^^4^^,^^6^^,^^7^

The recommendations for CCS interventions are less consistent and superficially consider that the procedures should be individualized and indicated only for high-risk patients.^6^^,^^8^ Welt et al.^8^ suggested reducing the number of procedures, with delays in elective cases, and dividing the staff into shifts to rotate professionals, with a focus on reducing the risk of staff contamination.

Those restrictive measures for stable patients provided a comfort zone in relation to reducing the spread of COVID-19; however, in objective terms, we observed that coronary heart disease care was not included, with regard to the good practices established before the pandemic.

In Brazil, the pandemic has dramatically changed medical care in several specialties with suspension of elective procedures and appointments. Most medical professionals advised their patients, either private health insurance clients or SUS clients, to postpone their elective procedures and to seek emergency rooms in case of severe symptoms of chest pain, dyspnea, etc. This guidance undoubtedly protects individuals who remain asymptomatic or oligosymptomatic from SARS-CoV-2 but exposes patients who may need urgent care to infection. Conversely, individuals who believe their symptoms are not so severe or who are more tolerant of pain may be affected by a reckless reflection. In such cases, delaying care may have serious consequences, including cardiac death at home.

Resolution number 2004 of March 18, 2020 issued by the Rio de Janeiro State Health Department^15^ suspended elective outpatient care services in public units but correctly maintained essential outpatient care services, including those of oncology and cardiology. Nonetheless, access to patients was affected and the resolution was not widely accepted. As a rule, SUS patients are more severe and have more risk factors. In those patients, there are great difficulties in separating those who are overall stable from those who may be affected by an acute condition requiring hospitalization.

It seems reasonable to us to preserve the capacity of hospital beds, avoiding unnecessary elective procedures in stable patients with significant comorbidities or in those whose post-intervention length of stay is more than 24 to 48 hours.However, despite being in line with most protective measures for patients and staff, we highlight that patients with CCS may require procedures and this warrants cautiously breaking the restrictive measures with no additional risks of exposure to the virus.

In this case series, an active search was made to select patients who were symptomatic, ischemic, and with multiple risk factors, thus preventing them from attending any type of appointment or going to an emergency room. We understand that such rapid action in the diagnosis and treatment of those patients was a measure that prevented a more severe outcome in terms of coronary events. Conversely, strict care and guidance provided in the appointment, reinforced isolation, procedural routines, and the attempt to reduce length of stay have minimized the risks of coronavirus infection.

In the health crisis triggered by COVID-19, there was no objective guidance on the performance of procedures in stable patients exactly because they were suspended or delayed. In our real-world experience, providing patients with suspected CAD with care based on clinical assessment and rapid knowledge of coronary anatomy should be done before any possible clinical instability. In this population, we clearly demonstrated that the risk of cardiac events to which patients were exposed was much greater than the possibility of having complications of the disease caused by the virus.

Although they do not determine that this is effectively the best approach, our data suggest a reflection so that care in catheterization laboratories during the pandemic is reassessed and not systematically suspended or delayed.

### Limitations

The main limitations are the small number of patients, the low-prevalence variables that require much larger samples, and the fact that this is a single-center study. In addition, under the guidance of the Hospital Infection Control Committee, we did not perform COVID-19 tests on any of the patients and staff because they were all asymptomatic at the time of the procedure and remained as such for at least 15 days. Despite the limitations, this study may encourage other services to generate multicenter observations and analyses with greater statistical robustness.

## Conclusions

As shown in this study, the performance of elective tests in patients with CCS was safe for both patients and professionals even during the pandemic, contrasting with most recommendations from other services. The study demonstrated that the anatomical angiographic evaluation revealed patients at high risk of morbidity and mortality, requiring interventions in those with complex lesions, thereby contributing to reduce the number of ACS in this population.
